# Musculotendinous Anatomy in Congenital Split Foot: Anatomical Description of a Rare Case and Literature Review

**DOI:** 10.3390/life16020189

**Published:** 2026-01-23

**Authors:** Vladimir Kenis, Dmitry Starchik, Alexander Kochish, Dmitry Busarin, Nino Abdiba

**Affiliations:** 1 H. Turner National Medical Research Center for Children’s Orthopedics and Trauma Surgery, 196603 St. Petersburg, Russia; 2 Department of Human Morphology, North-Western State Medical University named after I.I. Mechnikov, 191015 St. Petersburg, Russia; dmitrii.starchik@szgmu.ru (D.S.); busarindn@gmail.com (D.B.); 3 Vreden National Medical Research Center of Traumatology and Orthopedics, 195427 St. Petersburg, Russia; auk1959@mail.ru (A.K.); ninoabdiba@gmail.com (N.A.)

**Keywords:** split hand/foot malformation, anatomy, tendon loop

## Abstract

Background: Congenital split foot/hand is a rare limb anomaly. Although various surgical techniques have been described, detailed gross anatomical studies of soft tissue adaptation, particularly in the foot, are extremely rare. This study presents a detailed anatomical description of a case of severe bilateral split foot. Methods: A comprehensive dissection was performed on the lower limb of a 64-year-old male donor with bilateral split foot/hand. Results: Radiographic evaluation classified the deformity as Blauth type IV, characterized by the absence of the lateral cuneiform bone and severe hypoplasia/aplasia of the second and third metatarsals. Significant changes were revealed in the musculotendinous apparatus. The key finding was a unique tendon loop passing through the central cleft, formed by the tendon of the extensor digitorum longus and connecting with the tendons of the flexor digitorum longus and flexor hallucis longus. Conclusions: This study presents the first detailed macroscopic anatomical description of split foot, demonstrating that this congenital anomaly involves complex, structured tendon and muscle adaptations that extend beyond skeletal deficiencies alone. The discovery of a persistent tendon loop—previously reported only once in split hand—indicates asynchronous development of skeletal and soft tissue structures. These findings should be taken into account for surgical planning, emphasizing the need to identify and manage such abnormal soft tissue structures during reconstructive procedures.

## 1. Introduction

Congenital split hand/foot malformation (SHFM) is a rare and complex limb anomaly, predominantly genetic in origin. It manifests as a longitudinal deficiency of the hands and feet of varying severity—from shortening of one central ray to the complete absence of the first through fourth digits. Historically, numerous descriptive terms—such as ectrodactyly, lobster claw hand/foot, and pincer-like hand/foot—have been used to refer to this condition, reflecting its variable presentation and earlier attempts at phenotypic characterization [1]. In recent years, with expanding knowledge of its genetic causes, the term “congenital split hand/foot malformation” (SHFM) has become most prevalent in the literature [2]. The same term is used for the condition in the OMIM database of inherited traits.

The exact incidence of SHFM remains uncertain. In a landmark population-based study published in 1965, Arthur Barsky estimated the frequency of sporadic cases as 1 per 90,000 newborns and hereditary cases as 1 per 150,000 [3]. Despite methodological limitations when viewed from the perspective of modern population genetics, these figures have been repeatedly cited and remain a reference point in the literature. More recent studies give a higher incidence, in 8500–25,000 live births [4].

Genetic forms of SHFM most commonly follow an autosomal dominant inheritance pattern and are characterized by marked intrafamilial clinical variability. Sporadic cases may be caused by de novo mutations or chromosomal imbalances. When split hand/foot malformation occurs as part of a syndrome, the most frequently reported associations include EEC syndrome (ectrodactyly–ectodermal dysplasia–cleft lip/palate), lacrimo–auriculo–dento–digital (LADD) syndrome, the VACTERL association, and Cornelia de Lange and Smith–Lemli–Opitz syndromes [4,5].

In 2014, Sowińska-Seidler et al. summarized key genetic mechanisms, identifying seven genetic types of this pathology [6]. Most SHFM cases are inherited in an autosomal dominant manner (types 1, 3, 4, 5, and 7), but autosomal recessive (types 1 and 6) and X-linked inheritance (type 2) have also been reported. Knowledge of these subtypes facilitates the approach to genetic testing. A genetic diagnosis can currently be established in approximately 50–60% of cases. Exome and genome sequencing may further elucidate the genetic basis of this anomaly.

There are different approaches for the classification of SHFM [7]. The most detailed clinical–radiological classification of split feet was presented by Blauth and Borisch in 1990 [8]. The authors studied 45 split feet from their own patients and 128 from the literature, assessing radiographic anatomy. They developed a teratological series based on deformity severity: from a deepened central interdigital space to severe splitting with preservation of a single toe. These observations formed the basis of a classification dividing all variants into six groups according to the number of metatarsals. Types I and II involve split foot with minor defects and five metatarsals; the metatarsals are normal in type I and partially hypoplastic in type II. Type III has four metatarsals, type IV has three, and type V has two. Type VI represents split foot with preservation of a single (usually the fifth) toe.

Despite numerous publications on the surgical treatment of SHFM, the knowledge about the detailed anatomy of the soft tissues of the limbs in this condition is limited, because surgical approaches are mostly restricted to the hands and feet. To date, the only comprehensive anatomical description of a specimen with split hand has been published by Durand et al. (2009) [9]. In that study, a bilateral split hand was identified during anatomical dissection of a male donor at the Institute of Anatomy in Paris. The authors had no information regarding foot deformities or other malformations. Detailed anatomical investigation was performed on the right arm and forearm, while the left upper limb was plastinated. Both hands lacked the three central digits. The authors identified all forearm muscles with anatomical variations in attachment due to abnormal bone configuration. A key finding was the presence of flexor and extensor tendons of the missing digits, which fused together, forming a loop over the distal edges of the remaining carpal bones. The authors note encountering a similar phenomenon during reconstructive surgeries and recommend complete excision of this loop, paying particular attention to possible connections (commissures) with other tendons.

Notably, no comparable anatomical studies focusing specifically on split foot have been published to date, underscoring the uniqueness and relevance of the present investigation.

Numerous publications on the surgical treatment of congenital split hand/foot do not provide a complete picture of the anatomical changes in the forearm and hand, as surgical approaches do not allow for full tracing of tendon courses and muscle anatomy, being limited to the surgical field [5,10]. Nevertheless, knowledge of these features is important both for a general understanding of teratogenic patterns, which may contribute to a better understanding of embryogenesis, and for optimizing surgical treatment for patients with this pathology, highlighting the relevance of the present study.

Having access to a donor’s body of a patient with SHFM, we conducted a study aiming to analyze the anatomical features of a split foot specimen and identify possible patterns characteristic of this pathology by comparing our results with existing data from the scientific literature.

## 2. Materials and Methods

An anatomical study was performed on the body of a 64-year-old male with congenital splitting of both hands and both feet. Body length was 157 cm, weight 49 kg, mesomorphic constitution (body length-to-trunk length ratio index—31.2). The material had been preliminarily fixed in a 7% formalin solution. The study was conducted by professional anatomists at the Department of Human Morphology of the North-Western State Medical University named after I.I. Mechnikov after approval by the local ethics committee. In accordance with Federal Law of the Russian Federation No. 8-FZ “On Burial and Funeral Services” dated 12 January 1996, the donor provided written informed consent during life for the use of his body for educational and research purposes. Layer-by-layer dissection of the right leg and foot was performed with removal of the skin, subcutaneous tissue, and the deep (crural) fascia of the leg and foot, followed by meticulous isolation of each muscle and its tendons. Morphometric measurements of anatomical structures were obtained using a vernier caliper with an accuracy of 1 mm.

Foot and ankle radiographs in axial (plantar) and anteroposterior projections were taken using a UT 2000 X-ray machine (Philips Research, Eindhoven, The Netherlands) at a source-to-image distance of 90 cm at 50 kVp and 5 mAs.

## 3. Results

Gross examination revealed bilateral hypotrophy of the leg musculature, accompanied by shortening of the feet and flattening of the longitudinal arches on both the right and left sides. General morphometric parameters of the feet are provided in Table 1.

Analysis of weight-bearing anatomy demonstrated that the posterior bony loading point of the right foot projected onto the calcaneal tuberosity. The anterior loading points were located beneath the distal epiphyses of the medial and lateral metatarsals. Distances between the bony loading points of the right foot were as follows:

• Calcaneo–lateral: 130 mm.
• Calcaneo–medial: 135 mm.
• Lateral–medial: 50 mm.

At each weight-bearing site, rounded skin samples were excised to assess callus thickness. Measurements revealed the following values:

• Heel area: 1.6 mm.
• Medial loading point: 1.5 mm.
• Lateral loading point: 1.4 mm.

**Figure 1:**
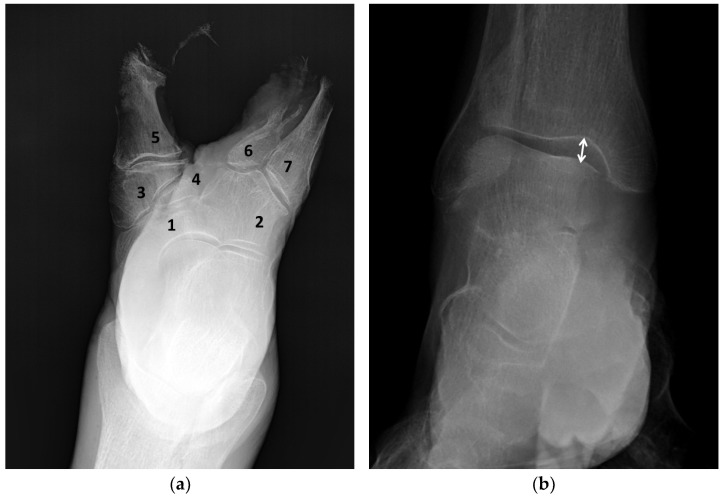
Radiographs of the right foot and ankle: (**a**)—plantar projection; (**b**)—anteroposterior projection: 1—navicular; 2—cuboid; 3—medial cuneiform; 4—intermediate cuneiform; 5—first metatarsal; bases and proximal portions of the diaphyses of the fourth (6) and fifth (7) metatarsals; widening of the joint space in the lateral ankle compartment (white arrow).

Radiographic examination of the right foot in the plantar view and ankle in the anteroposterior projection demonstrated preservation of the principal components of the hindfoot, including the talus, calcaneus, navicular, and cuboid bones, as well as the ankle joint (Figure 1). The medial and intermediate cuneiform bones were reduced in size and significantly deformed, while the lateral cuneiform was completely absent. Among the metatarsals, only the bases and proximal portions of the diaphyses of the first, fourth, and fifth metatarsals were present. The head of the first metatarsal was irregular in shape, while the fourth and fifth metatarsals lacked distal epiphyses. Overall, the radiographic findings corresponded to Blauth type IV split foot deformity.

Radiographic examination of the right ankle joint in the anteroposterior projection (Figure 1b) showed widening of the radiographic joint space in the lateral compartment, accompanied by relative narrowing of the talocalcaneal joint space on the medial side. An irregularly contoured soft tissue shadow consistent with a plantar callus was visualized along the lateral margin of the sole, extending from the calcaneal tuberosity to the level of the metatarsal bodies.

Layer-by-layer dissection of the right leg revealed hypotrophied ligaments, muscles, and their tendons. The origins of the muscles of the anterior, lateral, and posterior compartments of the leg and their topographic relationships with each other and leg bones showed no deviations from the typical for this body type. Distinctive features were identified exclusively in the number and topography of tendons passing distal to the ankle joint and in their sites of attachment to the bones of the foot.

On the anterior aspect of the ankle joint, two extensor retinacula were identified. The superior extensor retinaculum was the larger of the two, measuring 71 mm in height, whereas the inferior extensor retinaculum measured 35 mm.

**Figure 2:**
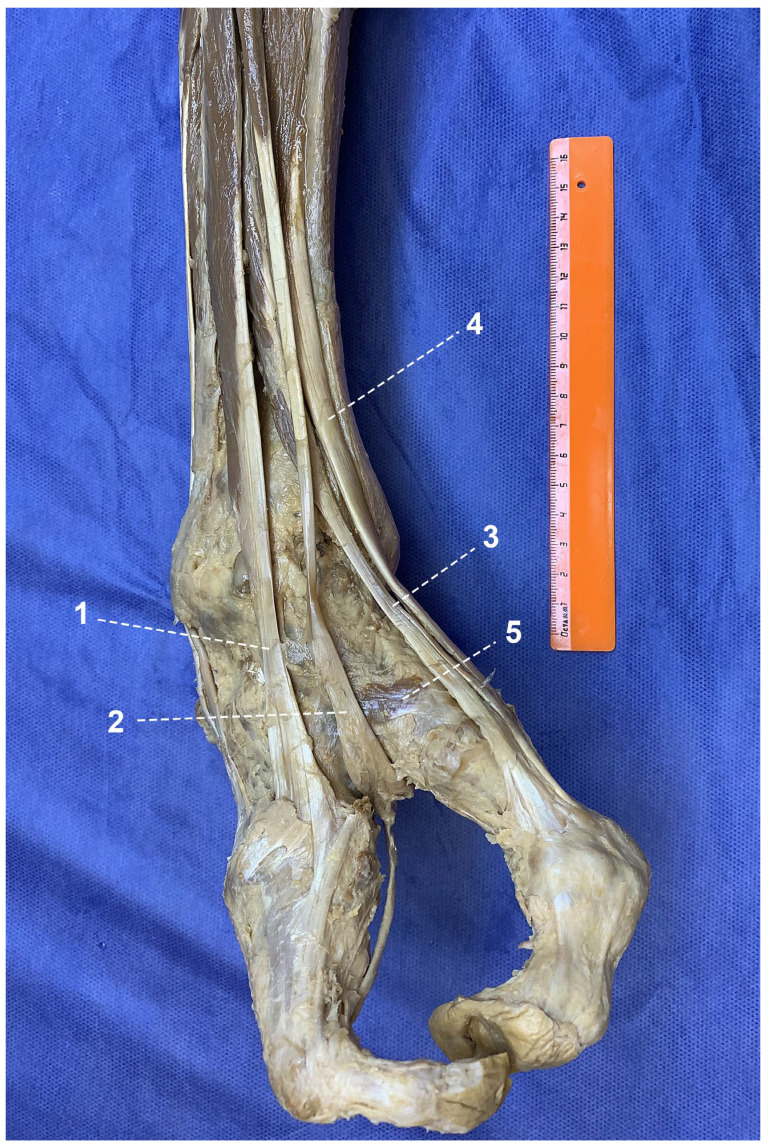
Muscles of the anterior compartment of the leg and dorsal aspect of the foot: 1—lateral belly of extensor digitorum longus; 2—medial belly of extensor digitorum longus; 3—extensor hallucis longus; 4—tibialis anterior; 5—extensor digitorum brevis and extensor hallucis brevis.

The most significant soft tissue findings of the present study involved the musculotendinous structures of the anterior compartment of the leg and dorsum of the foot. During dissection, the presence of two distinct muscle bellies of the extensor digitorum longus (EDL) was identified (Figure 2). The lateral portion of this muscle is inserted onto the base and superior surface of the fifth (lateral) metatarsal and continues distally along the lateral ray. In contrast, the tendon of the medial portion of the EDL was markedly shortened and passed between the two rays of the split foot, descending toward the plantar surface. At this level, it merged with the tendons of the flexor digitorum longus (FDL) and flexor hallucis longus (FHL), forming a well-defined tendinous loop. The peroneus tertius muscle was absent in the right foot. The tendon of the extensor hallucis longus (EHL) attached to the dorsal surface of the head of the first (medial) metatarsal and continued distally into the dorsal soft tissues of the medial ray, where osseous structures were largely absent. The insertion of the tibialis anterior tendon corresponded to the normal anatomical location.

During dissection of the dorsal aspect of the foot, rudimentary muscles of the dorsal compartment—the extensor hallucis brevis and extensor digitorum brevis—were identified on the dorsal surface at the junction between the medial and lateral rays.

**Figure 3:**
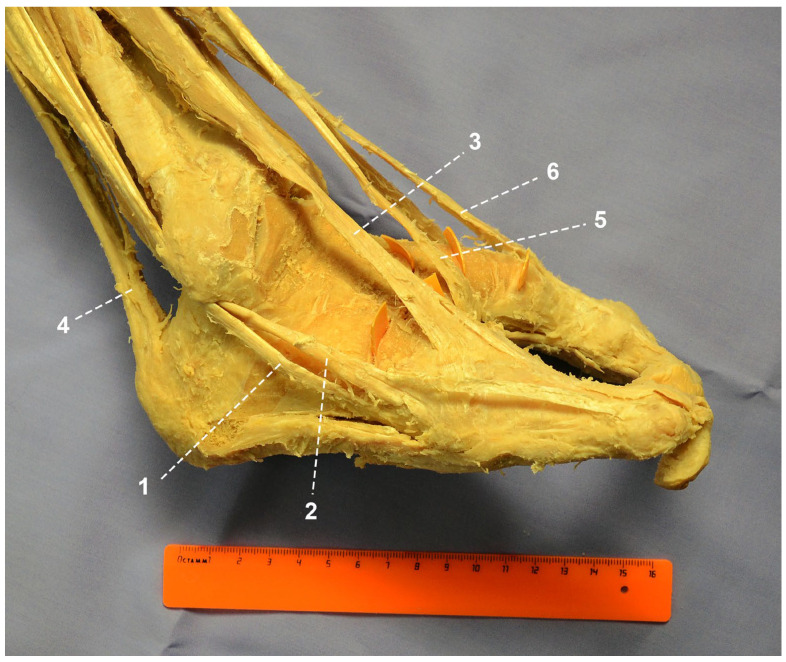
Attachment of peroneal tendons to the foot: 1—peroneus longus; 2—peroneus brevis; 3—lateral part of extensor digitorum longus; 4—Achilles tendon; 5—medial part of extensor digitorum longus; 6—extensor hallucis longus.

Within the lateral compartment, the peroneus longus tendon was shortened and inserted into the cuboid bone. The tendon of the peroneus brevis exhibited bifurcation: a broader and shorter branch inserted into the base of the fifth (lateral) metatarsal, whereas a longer and narrower branch continued distally onto the dorsal surface of the lateral ray (Figure 3).

Within the posterior compartment of the leg, structural abnormalities were confined to the deep muscle layer. The tibialis posterior tendon is inserted onto the inferior surface of the navicular bone. The tendons of the flexor digitorum longus (FDL) and flexor hallucis longus (FHL) converged and contributed to the formation of a tendinous loop by connecting with the medial tendon of the extensor digitorum longus (EDL).

**Figure 4:**
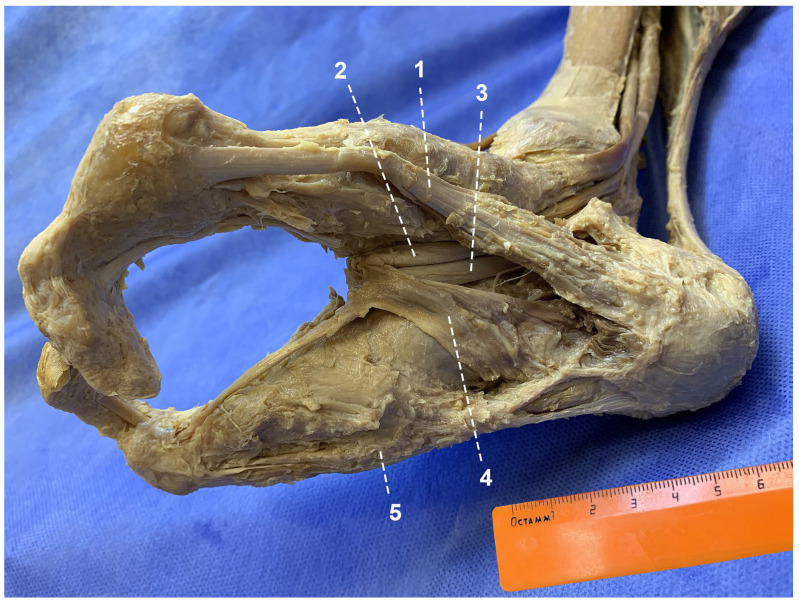
Muscles and tendons of the plantar aspect of the foot: 1—abductor hallucis; 2—flexor hallucis longus; 3—flexor digitorum longus; 4—flexor digitorum brevis; 5—abductor digiti minimi.

On the plantar aspect of the foot, the majority of the intrinsic plantar muscles were absent. These included flexor hallucis brevis; adductor hallucis; quadratus plantae; lumbricals; dorsal and plantar interossei; flexor digiti minimi brevis and opponens digiti minimi (Figure 4). Dissection of the split foot’s plantar surface revealed preservation of the abductor hallucis muscle on the medial ray, with a robust tendon attaching to the head of the first metatarsal. The flexor digitorum brevis was markedly hypoplastic. Its wide distal part connected with the extensor digitorum longus tendon, while a long, thin tendon continued onto the medial surface of the lateral ray. Along the lateral margin of the flexor digitorum brevis, the tendons of the flexor digitorum longus and flexor hallucis longus coursed distally. On the lateral aspect of the lateral ray, the abductor digiti minimi muscle was identified; its tendon inserted into the distal portion of the fifth metatarsal.

**Figure 5:**
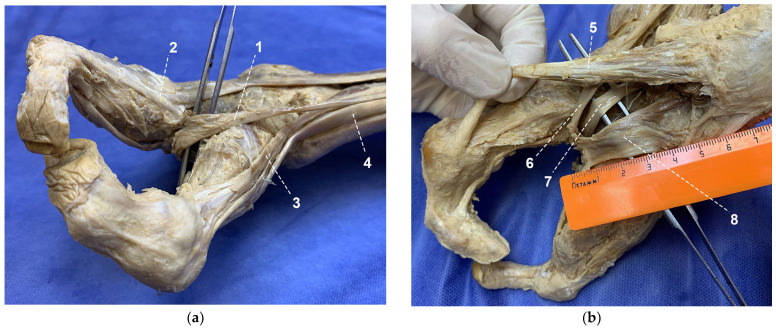
Formation of the tendinous loop on the split foot: (**a**) dorsal surface; (**b**) plantar surface; 1—medial part of extensor digitorum longus; 2—lateral part of extensor digitorum longus; 3—extensor hallucis longus; 4—tibialis anterior; 5—abductor hallucis; 6—flexor hallucis longus; 7—flexor digitorum longus; 8—flexor digitorum brevis.

Thus, in the examined split foot, many muscles were absent or hypoplastic. Owing to the absence of the second and third metatarsals and the phalanges of all toes, an unusual and well-defined tendinous loop was formed (Figure 5). This loop resulted from the convergence of the tendons of the long and short digital flexors, the flexor hallucis longus tendon, and the medial portion of the extensor digitorum longus tendon.

Table 2 summarizes the sites of muscle attachment in the split foot in comparison with the normal anatomical variant.

## 4. Discussion

Despite a long history of observation and surgical treatment of patients with congenital split hand/foot malformation, there is a gap in our knowledge about the residual anatomy of the soft tissues, related to the absent bony components of the hands and feet, first of all, the extrinsic muscles and their tendons. Our case provides valuable information in this field, confirming the existence of the muscle bellies of the extrinsic muscles of the absent toes, creating the loop resulting from the convergence of the tendons of the long and short digital flexors, the flexor hallucis longus tendon, and the medial portion of the extensor digitorum longus tendon.

In the only published description of an anatomical specimen of a split upper limb, forearm, and hand, Durand et al. traced the course of all forearm and hand muscle tendons and substantiated the fact that, despite the absence of metacarpals and digits, flexor and extensor tendons of the missing digits could be traced to the distal part of the cleft, where they formed a loop by connecting with each other [9]. This observation raised fundamental questions regarding the temporal and spatial relationship between skeletal and soft tissue development during intrauterine life.

The present case provides important additions to the overall picture of teratological changes in congenital limb anomalies. To our knowledge, this case is the second full description in the scientific literature of macroscopic changes in SHFM based on a complete anatomical study of the affected limb, and the first description of such changes in the foot. The most characteristic findings involved the musculotendinous structures associated with hypo- and aplastic rays, particularly the long flexors and extensors. The importance of this finding lies in the additional confirmation of the asynchronous formation of anatomical elements of the distal limb during embryogenesis and fetogenesis. These observations supplement the overall picture of phenotypic manifestations, which should later be correlated with the genotype in hereditary syndromes, including SHFM. In our case, genetic testing was not possible, but such studies are advisable in the future.

Preservation of mobility in the loop we discovered, formed by flexor and extensor tendons, likely maintained residual function in the muscle bellies of both muscles, as evidenced by the absence of significant hypotrophy. Knowledge of this fact may be essential when planning surgical interventions for complex split foot reconstructions—if additional reinforcement of specific muscle functions (flexors/extensors, invertors/evertors of the foot) is needed, relocation of these tendon attachment points could be used to achieve the corresponding goal. Furthermore, during surgery, it is important to remember that even in the absence of bony structures, tendons may be located within the cleft as a loop or may attach to adjacent digits.

The observed alterations in the anatomy of biarticular muscles —tibialis anterior and posterior, peroneus longus and brevis—are also highly interesting. The normal anatomy of these muscles, their distal attachment points, and the direction of their tendons determine their primary role in the propelling and balancing functions of the foot. These changes must also be carefully considered during surgical planning to avoid secondary deformities.

SHFM is a rare and complex developmental anomaly that frequently requires surgical intervention. Advances in molecular genetics have led to the identification of seven genetic subtypes of SHFM, improving our understanding of inheritance patterns and associated malformations [11]. The condition is genetically heterogeneous and may be caused by chromosomal rearrangements, as well as point mutations [12]. As a result of marked genetic heterogeneity, variable expressivity, and incomplete genotype–phenotype correlation, molecular diagnosis of isolated SHFM remains challenging [13].

Limb function in SHFM can be impaired due to anatomical changes, although self-care may be minimally affected as children adapt notably well. However, the cosmetic defect can have a significant psychological impact. Surgical treatments aim not only to improve function but also to achieve a more aesthetically satisfactory limb appearance.

The main limitations of this study include the absence of genetic analysis, as donor documentation did not permit such investigation. In addition, the body was preserved using a formaldehyde solution, which precludes technically routine genetic testing. In addition, for this publication, we did not conduct an atomic study of the contralateral foot and hands, which is planned to be carried out in the future.

## 5. Conclusions

Thus, the present study demonstrates that changes in split foot involve not only aplasia or hypoplasia of bones and joints but also characteristically associated alterations in muscles and tendons. The actual bone and joint anomaly of the forefoot can primarily lead to the impairment of the supportive function, while the abnormal tendon anatomy can impair the propelling and balancing functions.

In the surgical treatment of congenital malformations, including SHFM, a clear understanding of possible anatomical features is crucial for optimal planning and execution. The findings of this study significantly expand current anatomical understanding of split foot deformities and highlight reproducible patterns of musculoskeletal reorganization.

## Figures and Tables

**Table 1:** General parameters of split feet (mm).

Parameter	Right Foot	Left Foot
Length	176	184
Width	87	92
Height	58	63

**Table 2:** Muscle attachment sites on normal and split foot.

Muscle	Attachment Site on Normal Foot	Attachment Site on Split Foot
M. tibialis anterior	Plantar surface of medial cuneiform, base of 1st metatarsal	Plantar surface of medial cuneiform, base of 1st metatarsal
M. extensor digitorum longus	Middle and distal phalanges of toes II–V	Medial part: tendinous loop; Lateral part: distal epiphysis of 5th metatarsal
M. extensor hallucis longus	Distal phalanx of great toe (I)	Head of the 1st metatarsal (I)
M. peroneus longus	Tuberosity of 1st metatarsal, base of 2nd metatarsal, medial cuneiform	Cuboid bone
M. peroneus brevis	Tuberosity of 5th metatarsal (V)	Base of the 5th metatarsal (V)
M. triceps surae	Calcaneal tuberosity	Calcaneal tuberosity
M. plantaris	Calcaneal tuberosity	Calcaneal tuberosity
M. flexor digitorum longus	Distal phalanges of toes II–V	Tendinous loop
M. tibialis posterior	Navicular tuberosity, medial, intermediate, and lateral cuneiforms	Navicular tuberosity
M. flexor hallucis longus	Distal phalanx of great toe (I)	Tendinous loop
M. flexor digitorum brevis	Middle phalanges of toes II–V	Tendinous loop
M. abductor hallucis	Proximal phalanx of great toe (I)	Head of the 1st metatarsal (I)
M. abductor digiti minimi	Tuberosity of 5th metatarsal (V), proximal phalanx of 5th toe (V)	Distal epiphysis of 5th metatarsal (V)

## Data Availability

The original contributions presented in this study are included in the article. Further inquiries can be directed to the corresponding author.

## Abbreviations

The following abbreviations are used in this manuscript:

SHFM	Split Hand/Foot Malformation
AER	Apical Ectodermal Ridge
OMIM	Online Mendelian Inheritance in Man
EDL	Extensor Digitorum Longus
FDL	Flexor Digitorum Longus
FHL	Flexor Hallucis Longus
EHL	Extensor Hallucis Longus
